# Key metabolic parameters change significantly in early breast cancer survivors: an explorative PILOT study

**DOI:** 10.1186/s12967-019-1850-2

**Published:** 2019-04-01

**Authors:** Stine Overvad Fredslund, Claus Højbjerg Gravholt, Britt Elmedal Laursen, Anders Bonde Jensen

**Affiliations:** 1 Department of Oncology, Aarhus University Hospital, Nørrebrogade 44, 8000 Aarhus C, Denmark; 2 Department of Endocrinology and Internal Medicine MEA, Aarhus University Hospital, Palle Juul Jensens Blvd 99, 8200 Arhus N, Denmark; 3 Department of Molecular Medicine (MOMA), Aarhus University Hospital, Brendstrupgårdsvej 100, 8200 Aarhus N, Denmark; 4 Department of Biomedicine, Aarhus University, Wilhelm Meyers Allé 3, 8000 Aarhus C, Denmark

**Keywords:** Breast cancer, Chemotherapy, Long-term side effects, Metabolic syndrome, Cardiovascular risk, Body composition, Dyslipidemia

## Abstract

**Background:**

With increasing number of breast cancer survivors, more attention is drawn to long-term consequences of curative cancer treatment. Adjuvant treatment of breast cancer patients is associated with several unfavorable medical conditions, including dyslipidemia, insulin resistance, and obesity, potentially leading to cardiovascular disease and/or the metabolic syndrome. The aim of this explorative study is to investigate metabolic side effects of adjuvant treatment in breast cancer patients.

**Methods:**

A cohort of 13 premenopausal and 20 postmenopausal women with early stage breast cancer were extensively examined prior to, immediately after and 1 year after ended adjuvant chemotherapy and compared with healthy controls (N = 36) matched by age and menopausal status. Repeated examinations included: anthropometric measures, DEXA scans, 24-h blood pressure measurements, and blood samples [high sensitivity CRP, lipid profile and glucose metabolism, including homeostatic model assessment (HOMA)].

**Results:**

At baseline, breast cancer patients were similar to healthy controls regarding all measures. From baseline to 1-year post-treatment specific components of the metabolic syndrome increased significantly in premenopausal breast cancer patients; body fat (P = 0.01), triglycerides (P = 0.03), waist circumference (P = 0.008) and diastolic blood pressure (P = 0.04). In postmenopausal patients, waist circumference also increased significantly (P = 0.03), and High density lipoprotein (HDL) cholesterol decreased significantly (P = 0.05).

**Conclusions:**

Specific components of the metabolic syndrome changed significantly during chemotherapy in early stage breast cancer patients. After 1 year, several key parameters remained pathologically changed. Premenopausal breast cancer patients seemed to be especially prone to develop these unfavorable changes.

*Trial registration* ClinicalTrial.gov, registration number NCT02652975. Registered 15 December 2015—Retrospectively registered, https://clinicaltrials.gov/.

## Background

With improved and intensified treatment, an increasing number of women survive breast cancer (BC) [1]. Women going through a course of adjuvant treatment for BC potentially receive several cardiotoxic agents, as anthracyclines, cyclophosphamide, and trastuzumab, and agents that can interfere with the metabolic and hormonal balance of the women, e.g. aromatase inhibitors (AIs), selective estrogen receptor modulators (SERMs) and corticosteroids.

Several studies have found that BC survivors are at increased risk of cardiovascular disease (CVD) compared with matched controls [2, 3]. In 2011, Patnaik et al. [4] showed that among 63,566 BC patients, CVD was the primary cause of death (15.9%), followed closely by BC (15.1%), and a recent prospective cohort study concludes that women with a previous BC have a 1.77 times higher risk of CVD mortality than women without BC [5]. Likewise, diabetes and the metabolic syndrome are more prevalent among BC survivors compared to age matched controls [6–8].

Metabolic syndrome (MetS) is a cluster of disorders including hypertension, type II diabetes, dyslipidemia and obesity. Especially visceral adiposity is considered to be a major driving force in the development of MetS [9].

In both pre- and postmenopausal BC patients, estrogen levels are found to decrease during adjuvant chemotherapy [10], and chemotherapy given to premenopausal women can lead to premature menopause (permanently or transient). The most common metabolic disorders in menopause include dyslipidemia, obesity, impaired glucose tolerance, insulin resistance, hyperinsulinemia, and type II diabetes [11, 12]. The risk of type II diabetes increases after menopause, with earlier onset of menopause leading to higher risk [13].

Both AIs and SERMs can induce menopausal symptoms [14, 15]. These symptoms can indirectly lead to change of life style, inactivity, weight gain, and muscle atrophy, which will increase the risk of pathological metabolic changes related to MetS. Another side effect directly associated to hormonal therapy is dyslipidemia. Tamoxifen is known to have a cardioprotective effect, including reducing total cholesterol levels and low-density lipoprotein cholesterol (LDL) [16], but at the same time it can induce severe hypertriglyceridemia [17]. A significantly higher risk of diabetes has also been found in older BC survivors treated with tamoxifen compared with no endocrine therapy [18].

The aim of this prospective explorative observational study was to determine metabolic changes during adjuvant treatment, and the 1st year after, in both pre- and postmenopausal BC patients, in order to highlight the burden of metabolic and cardiovascular comorbidity caused by adjuvant cancer treatment.

## Methods

### Participants

**Fig. 1:**
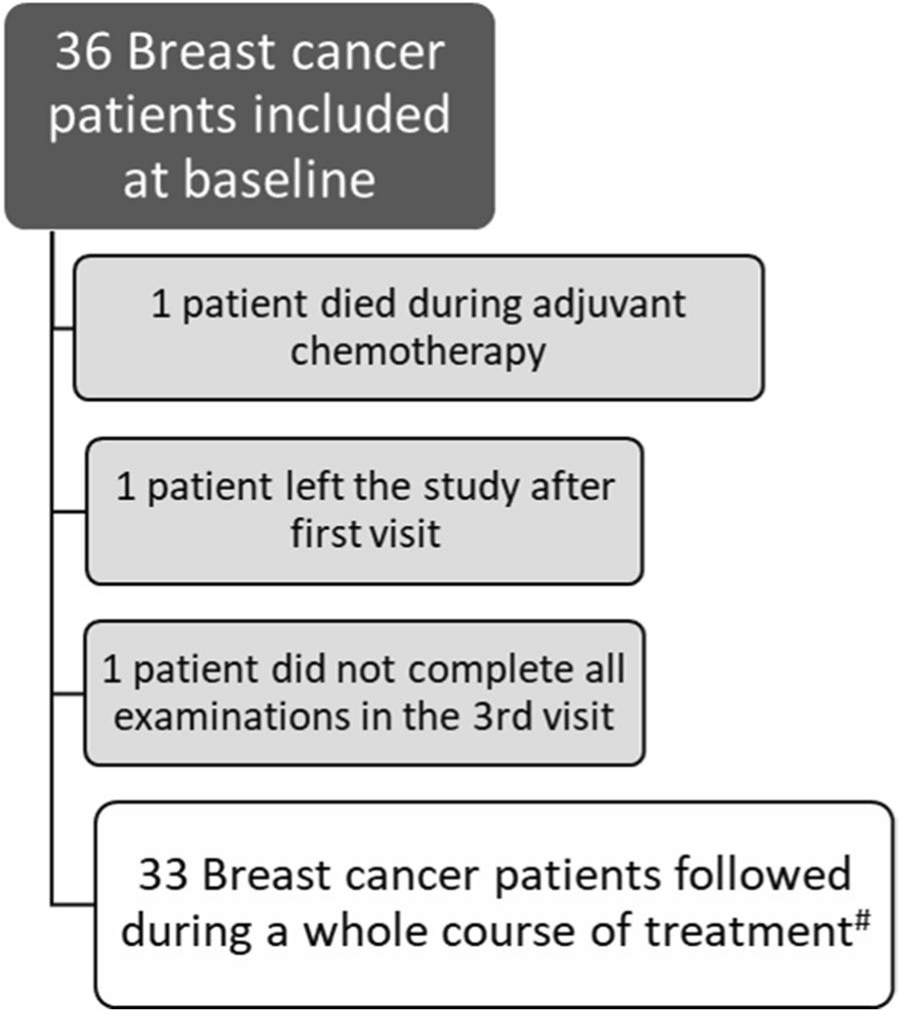
Enrolment of breast cancer patients. ^#^Before start of and until 1 year after completed chemotherapy

We recruited pre- and postmenopausal women with newly diagnosed, early-stage BC from the Department of Oncology at Aarhus University Hospital (AUH). Participant were recruited between September 2015 and December 2016. The last participant visit was completed 22th of June 2018. A research nurse screened all new patients with primary BC for the following inclusion criteria; (1) newly diagnosed primary BC, (2) assigned to receive adjuvant chemotherapy after lumpectomy or mastectomy, (3) age ≥ 18 years. Exclusion criteria were metastatic disease, previous or present treatment with chemotherapy, and/or pregnancy. We included 36 BC patients at baseline immediately after surgery and before start of adjuvant chemotherapy. One of the 36 women died after having received two cycles of chemotherapy (epirubicin and cyclophosphamide), one woman chose to leave the study after the first visit, and one woman failed to complete the investigational program at the third visit, leaving 33 participants for the present analyses (Fig. 1). None of the patients developed metastatic disease during the study period. Patient characteristics are presented in Table 1.

**Table 1:** Age, menopausal stage, tumor characteristics and treatment

	Premenopausal^a^	Postmenopausal^a^	All
Number	13	20	33
Mean age at baseline	46.9 (36–54)	58.4 (50–71)	53.8 (36–71)
ER status			
Positive	12	15	27
Negative	1	5	6
Tumor size (cm)			
0.1–2.0	10	10	20
2.1–5.0	2	10	12
> 5.0	1	0	1
Histological grade			
I	3	1	4
II	4	7	11
III	6	10	16
Unknown	0	2	2
HER 2 status			
Normal	9	17	26
Overexpression	4	3	7
LN involvement			
No	5	11	16
Yes	8 (1, 11)	9 (1, 2)	17
Laterality			
Right	6	11	17
Left	6	9	15
Bilateral	1	0	1
Pathology			
IDC	9	17	26
ILC	3	1	4
Other	1	2	3
Proliferation grade			
< 35	7	8	15
35–70	5	9	14
> 70	1	2	3
Unknown	0	1	1
Treatment			
Chemotherapy			
Epirubicin	13	20	33
Cyclophosphamide	13	20	33
Docetaxel	4	4	8
Paclitaxel	9	16	25
Supplementary adjuvant treatment			
Letrozole	0	12^b^	12
Tamoxifen	9^b^	1^c^	10
Trastuzumab	1	1	2
Tamoxifen + trastuzumab	3	0	3
Letrozole + trastuzumab	0	2	2
Only chemotherapy	0	4	4

Four women (50%) receiving EC/Doc were reduced in dose because of side effects. One of these women was premenopausal

11 women (44%) receiving EC/Pac were reduced in dose because of side effects. All of these women were postmenopausal

One of the women receiving Paclitaxel had received one cycle of docetaxel before continuing with two cycles of paclitaxel

*ER* estrogen receptor, *HER* human epidermal growth factor receptor, *LN* lymph node

^a^Menopausal status defined as: (A) premenopausal: menostasis < 12 month; hysterectomy and age < 55 years; cyclic hormonal treatment and age < 55 years. (B) Postmenopausal: all other women than the premenopausal and all oophorectomized

^b^Two patients assigned for letrozole and one assigned for tamoxifen discontinued within the 1st year because of side effects

^c^One postmenopausal women switched from letrozole to tamoxifen within the 1st year because of side effects

For each patient, one healthy control matched by age and menopausal status was included. The controls were recruited in part by means of the homepage www.forsogsperson.dk (10.01.19), by means of paper postings in and around the local hospital, and by means of a post on https://www.facebook.com/stine.o.fredslund/posts/10154604383376131 (10.01.19). The post was shared more than 100 times in 24 h, and 31 women volunteered. Of these, 16 women matched a case and was included in the study. Exclusion criterias for the healthy controls were pregnancy and previous or present treatment with chemotherapy.

### Methodology

All study measurements were performed on a single day, followed by a 24-h blood pressure measurement. The patients were examined three items; at baseline right after surgery (lumpectomy or mastectomy) and before start of chemotherapy (T0), after the final treatment (within 2 weeks after the completion of chemotherapy) (T1), and 1 year (11–13 months) after completed chemotherapy (T2). Healthy controls were only examined once.

### Laboratory tests

Blood glucose levels were measured in venous plasma using a hexokinase-glucose-6-phosphate dehydrogenase assay (Abbott, Chicago, IL, USA). Serum insulin was measured by ELISA utilizing dual-monoclonal antibodies (ALPCO, Salem, NH, USA), and HbA1c levels by cation exchange HPLC (G8 analysis, Tosoh Bioscience. Tokyo, Japan).

Using lithium-heparin total cholesterol, LDL, High density lipoprotein (HDL), and triglycerides were determined by means of chromogenic methods, before 29th of October 2016 on the Cobas 6000c analyzer (Roche) and after 29th of October 2016 on the Advia Chemistry XPT (Siemens). LDL is an estimate from the formula: LDL = total cholesterol − HDL − (0.45 × Triglyceride). A thorough validation was made after changing the equipment, and no clinical significant deviations were found in the median of the parameters.

By means of the homeostatic model assessment (HOMA2) calculator, insulin resistance (HOMA-IR) and beta cell function (HOMA-β) were calculated [19]. The calculator is set up to process insulin levels from 20 to 400 pmol/L and fasting glucose levels from 3.5 to 25 mmol/L. Some participants had insulin levels below 20 pmol/L, and in these cases, we used 20 pmol/L as the default value for calculations. Estradiol was measured with liquid chromatography tandem mass spectrometry (LC–MS/MS). Follicle-stimulating hormone (FSH) and luteinizing hormone (LH) was measured by means of immunochemistry testing on the Cobas 6000 analyzer. Using lithium-heparin high sensitivity C-reactive protein (hs-CRP) were determined by means of immunochemical reaction on the Cobas 6000 analyzer. From serum CD163 and CD206 was extracted and analyzed by means of ELISA on the BEP2000 Advance analyzer.

All blood samples were analyzed at the Research Laboratory of the Department of Renal Medicine at Aarhus University Hospital, except for serum insulin, which was analyzed at the Research Laboratory of the Department of Endocrinology and Internal Medicine at Aarhus University Hospital, and hs-CRP that was analyzed at the Department of Clinical Biochemistry at Aarhus University Hospital.

### Dual-energy X-ray absorptiometry (DEXA) scan

Body fat percentage, lean body mass (LBM) and bone mineral density (BMD) of the total body and major sub regions were measured with whole body DEXA. A trained laboratory technician at the Research Laboratory of the Department of Endocrinology and Internal Medicine at Aarhus University Hospital performed all DEXA scans using a Hologic (Bedford, MA, USA) whole-body scanner model Discovery W.

### 24-h blood pressure measurement

Twenty-four hours systolic/diastolic blood pressure values were recorded with a device that inflates and measures every 20th minute. The monitoring device (Spacelabs 90217, Issaquah, Wash., USA) was calibrated, and placed with an appropriately sized cuff on the upper arm on the opposite site compared to the operated breast. The device was worn for 24 h, while the participants continued their normal activities.

### Clinical examination

Clinical examination included auscultation of heart and lungs as well as assessment of general condition and signs of congestive heart disease. Anthropometric data were obtained (weight, height, Body mass index (BMI), waist- and hip circumference), the latter were measured using a fabric measuring tape around the waist (centered at the navel) and hip (centered on the greater trochanter).

Information about age, age at menarche and menopause, number of full-born children and breast-feeding length, as well as lifestyle habits (alcohol consumption and smoking status; if smoking, the smoking index [https://www.smokingpackyears.com/, (10.01.19) was calculated] was obtained, as were data concerning comorbidity and prescribed medication.

MetS was defined as follows; three or more of the following five criteria must be met: HDL < 1.3, triglycerides ≥ 1.7, waist circumference > 88 cm, fasting glucose ≥ 6.1, blood pressure ≥ 130/85.

### Statistical analysis

Data on both BC patients and healthy controls were analyzed separately in two groups; pre- and postmenopausal women. For postmenopausal women, separate analysis were also performed without women receiving statins and/or metformin, to examine whether these medications had an impact on outcome.

Baseline measurements of patients were compared with measurements on healthy controls using Student’s two-sample t-test for non-paired data, and for non-parametric data, Wilcoxon rank sum test. To examine the change in relevant parameters during follow up, we used paired t-tests comparing the baseline levels (T0) with the levels at the two different time points during follow up (T1 and T2). Triglycerides, BMI, hs-CRP, insulin, HOMA-values, FSH, LH, and estradiol was assessed as non-normally distributed [19]. For triglycerides, BMI, hs-CRP, FSH, LH, and estradiol non-parametric tests were performed and for insulin and HOMA-values, we used logarithmic transformed data.

All analyses were performed using Stata software, version 14.1 (Stata Corp., College Station, TX). A P < 0.05 was considered statistically significant.

## Results

We enrolled 36 BC patients in this study and 33 of these completed all investigations. Hereof, 13 patients were premenopausal and 20 were postmenopausal at baseline (Table 1). In addition, we enrolled 36 healthy controls (15 premenopausal women and 21 postmenopausal women).

All patients received standard adjuvant chemotherapy. 13 received Tamoxifen, 14 received Letrozol and seven received Trastuzumab (some of these combined). Four patients received chemotherapy alone (Table 1). The postmenopausal women were additionally offered treatment with bisphosphonates.

At baseline the majority of patients received no medication for co-morbidities, however seven BC patients (one premenopausal), and five controls, all postmenopausal, were treated with antihypertensive medicine. Two postmenopausal BC patient received statins at baseline, and three postmenopausal women in the control group were treated with statins. During the follow up period one postmenopausal BC patient started metformin.

After ended chemotherapy all postmenopausal BC patients were offered Zoledronic acid to reduce risk of bone metastases and increase overall survival [20]. One postmenopausal patient chose not to receive this treatment, because of fear of side effects, and one did not receive the treatment because of uncertainty about the menopausal status.

### Metabolic profile of breast cancer patients compared to healthy controls

At baseline, the BC patients and the healthy controls had similar metabolic profiles (Tables 2 and 3). No differences in female hormones were seen at baseline between postmenopausal BC patients and their respective controls. In premenopausal BC patients, FSH levels were significantly lower at baseline compared to their controls, while levels of LH and estradiol were similar in the patient and control group.

**Table 2:** Metabolic profile and females sex hormones of premenopausal patients at baseline (T0) compared to premenopausal healthy controls

	Premenopausal controls (n = 15)	Premenopausal BC patients (T0) (n = 13)	P-value^c^
BMI (kg/m^2^)^b^	***24.4*** [22.4; 26.0]	***25.7*** [22.6; 29.0]	*0.4*
Waist (cm)^a^	***84.6*** ± 9.93	***84.8*** ± 6.5^e^	*0.9*
Body fat (%)^a^	***32.3*** ± 5.70	***34.1*** ± 7.0	*0.4*
Lean body mass (kg)^a^	***44.2*** ± 4.88	***44.8*** ± 4.93	*0.6*
Bone mineral density (g/cm^2^)	***1.20*** ± 0.08	***1.17*** ± 0.05	*0.2*
Total cholesterol (mmol/L)	***5.03*** ± 0.94	***5.22*** ± 0.94	*0.6*
LDL (mmol/L)^a^	***3.06*** ± 0.86	***2.93*** ± 0.96	*0.7*
HDL (mmol/L)^a^	***1.52*** ± 0.39	***1.78*** ± 0.47	*0.1*
Triglyceride (mmol/L)^b^	***0.8*** [0.7; 1.36]	***1.10*** [0.88; 1.3]	*0.2*
Glucose (mmol/L)^a^	***5.2*** ± 0.38	***5.5*** ± 0.47	*0.1*
Insulin (pmol/L)^b^	***27.5*** [21.8; 34.6]	***36.5*** [25.8; 51.6]	*0.1*
HbA1c (mmol/mol)^a^	***35.1*** ± 3.13	***35.3*** ± 4.15	*0.9*
Systolic blood pressure (mmHg)	***115*** ± 10.6	***112*** ± 11.8	*0.5*
Diastolic blood pressure (mmHg)	***72.3*** ± 7.12	***71.0*** ± 8.47	*0.7*
Hs-CRP (mg/L)^b^	***0.63*** [0.6; 1.2]	***1.04*** [0.68; 2.45]	*0.1*
CD163 (mg/L)	***1.54*** [1.40; 2.04]	***1.66*** [1.27; 2.10]	*0.9*
CD206 (mg/L)	***0.20*** [0.15; 0.24]	***0.21*** [0.17; 0.25]	*0.6*
MetS^f^	***2 (13%)***	***0 (0%)***	*0.2* ^d^
FSH (IU/L)	***8.4*** [5.82; 31.6]	***3.9*** [3.0; 22.1]	0.03*
LH (IU/L)	***9.9*** [7.39; 25.5]	***4.7*** [3.0; 36.1]	*0.1*
Estradiol (pmol/L)	***338*** [119; 701]	***471*** [124; 752]	*0.6*

The italic values reflect P-values, and the bolditalic values reflects means/medians

*BMI* body mass index, *LDL* low-density lipoprotein, *HDL* high-density lipoprotein, *HbA1c* hemoglobin A1c, *Hs-CRP* high sensitivity C-reactive protein, *MetS* metabolic syndrome, *FSH* follicle-stimulating hormone, *LH* luteinizing hormone

* Statistical significant

^a^Mean

^b^Median

^c^Two sample T-test for means and Wilcoxon rank sum test for medians

^d^Pearson chi^2^ test

^e^n = 12

^f^At least 3 of the following 5: HDL < 1.3, triglycerides ≥ 1.7, waistline > 88 cm, fasting glucose ≥ 6.1, blood pressure ≥ 130/85

**Table 3:** Metabolic profile and females sex hormones of postmenopausal patients at baseline (T0) compared to postmenopausal healthy controls

	Premenopausal controls (n = 21)	Premenopausal BC patients (T0) (n = 20)	P-value^c^
BMI (kg/m^2^)^b^	***26.2*** [23.1; 28.2]	***26.3*** [22.7; 30.8]	*0.6*
Waist (cm)^a^	***89.0*** ± 10.3	***88.7*** ± 11.1^e^	*1.0*
Body fat (%)^a^	***38.1*** ± 6.57	***38.5*** ± 5.77	*0.8*
Lean body mass (kg)^a^	***42.5*** ± 6.18	***43.5*** ± 6.13	*0.6*
Bone mineral density (g/cm^2^)	***1.06*** ± 0.08^f^	***1.08*** ± 0.11	*0.6*
Total cholesterol (mmol/L)	***5.72*** ± 0.89	***5.56*** ± 0.83	*0.5*
LDL (mmol/L)^a^	***3.39*** ± 0.76	***3.27*** ± 0.90	*0.6*
HDL (mmol/L)^a^	***1.81*** ± 0.49	***1.73*** ± 0.52	*0.6*
Triglyceride (mmol/L)^b^	***1.1*** [0.9; 1.2]	***1.15*** [0.9; 1.2]	*0.6*
Glucose (mmol/L)^a^	***5.6*** ± 0.74	***5.9*** ± 0.61	*0.2*
Insulin (pmol/L)^b^	***37.3*** [28.2; 49.3]	***41.1*** [32.0; 52.8]	*0.6*
HbA1c (mmol/mol)^a^	***38.8*** ± 3.43	***37.0*** ± 4.64	*0.2*
Systolic blood pressure (mmHg)	***117*** ± 12.1	***119*** ± 11.3	*0.7*
Diastolic blood pressure (mmHg)	***74.5*** ± 7.19	***72.4*** ± 5.87	*0.3*
Hs-CRP (mg/L)^b^	***0.93*** [0.6; 1.77]	***1.43*** [0.76; 3.9]	*0.06*
CD163 (mg/L)	***1.77*** [1.58; 2.16]	***1.93*** [1.77; 2.08]	*0.3*
CD206 (mg/L)	***0.22*** [0.17; 0.26]	***0.23*** [0.18; 0.29]	*0.5*
MetS^g^	***3 (14%)***	***5 (25%)***	*0.4* ^d^
FSH (IU/L)	***78*** [57.8; 92]	***74.5*** [62.2; 98.4]	*0.7*
LH (IU/L)	***39*** [27.9; 49.1]	***41.5*** [36; 44.9]	*1.0*
Estradiol (pmol/L)	***16*** [15; 27.4]	***15*** [15; 22.7]	*0.6*

The italic values reflect P-values, and the bolditalic values reflects means/medians

*BMI* body mass index, *LDL* low-density lipoprotein, *HDL* high-density lipoprotein, *HbA1c* hemoglobin A1c, *Hs-CRP* high sensitivity C-reactive protein, *MetS* metabolic syndrome, *FSH* follicle- stimulating hormone, *LH* luteinizing hormone

* Statistical significant

^a^Mean

^b^Median

^c^Two sample T-test for means and Wilcoxon rank sum test for medians

^d^Pearson chi^2^ test

^e^n = 17

^f^n = 20

^g^At least 3 of the following 5: HDL < 1.3, triglycerides ≥ 1.7, waistline > 88 cm, fasting glucose ≥ 6.1, blood pressure ≥ 130/85

### Changes in body composition, metabolic parameters and female hormones in premenopausal BC patients

**Fig. 2:**
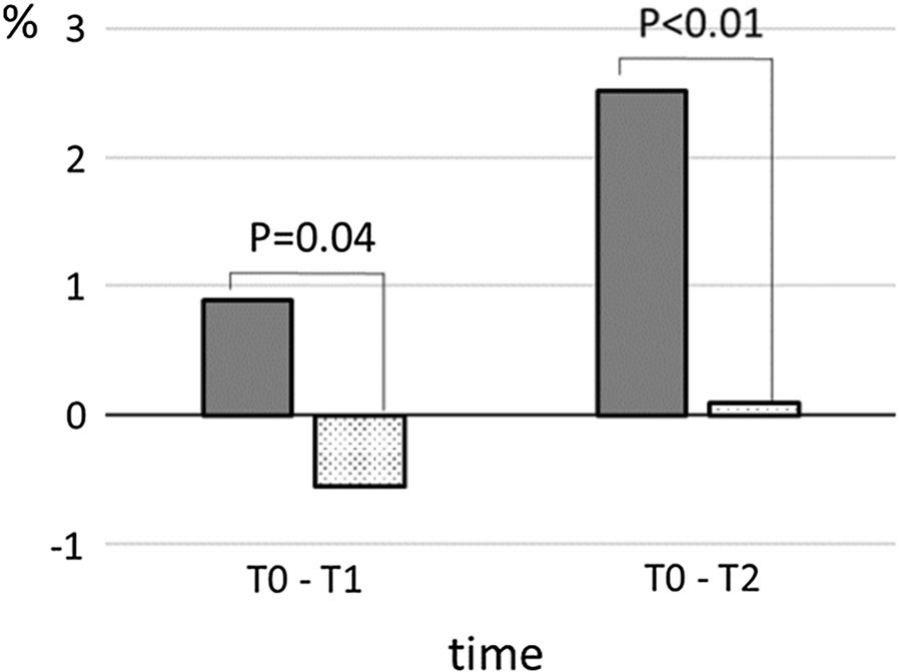
Change in body fat from T0 to T1 and from T0 to T2 depending on menopausal status. Dotted area, postmenopausal; grey-shaded area, premenopausal

Immediately after chemotherapy (T1), fat gain was evident among the premenopausal women (Fig. 2). Body fat and waist circumference increased statistically significant from T0 to T2 (Table 4), while there was no change in weight, hip circumference, lean body mass and bone mineral density.

**Table 4:** Changes in parameters related to the metabolic syndrome and females sex hormones in *premenopausal* BC patients during adjuvant anticancer treatment

	Mean/median			Paired t-test/signed rank^c^	
	T0	T1	T2	T0 vs. T1	T0 vs. T2
Weight (kg)^a^	***73.3*** ± 9.56	***74.3*** ± 9.31	***74.7*** ± 10.7	*0.1*	*0.1*
BMI (kg/m^2^)^b^	***25.7*** [22.5; 29.0]	***25.4*** [23.0; 29.1]	***25.2*** [23.0; 29.2]	*0.1*	*0.2*
Waist (cm)^a, g^	***84.0*** ± 6.07	***85.5*** ± 6.11	***87.7*** ± 7.91	*0.2*	*0.008* ^e^
Body fat (%)^a^	***34.1*** ± 7.00	***35.0*** ± 7.48	***36.7*** ± 5.87	*0.06*	*0.01* ^e^
Lean body mass (kg)^a^	***44.8*** ± 4.93	***45.1*** ± 5.01	***43.9*** ± 4.45	*0.4*	*0.1*
Bone mineral density (g/cm^2^)	***1.17*** ± 0.05	***1.16*** ± 0.06	***1.15*** ± 0.05	*0.5*	*0.08*
Total cholesterol (mmol/L)	***5.22*** ± 0.94	***5.51*** ± 1.28	***5.22*** ± 1.01	*0.3*	*1.0*
LDL (mmol/L)^a^	***2.93*** ± 0.96	***3.44*** ± 1.18	***2.77*** ± 0.87	*0.06*	*0.5*
HDL (mmol/L)^a^	***1.78*** ± 0.47	***1.42*** ± 0.43	***1.76*** ± 0.49	*0.003* ^e^	*0.8*
Triglyceride (mmol/L)^b^	***1.10*** [0.88; 1.3]	***1.20*** [1.0; 1.76]	***1.20*** [0.80; 2.10]	*0.05* ^e^	*0.03* ^e^
Glucose (mmol/L)^a^	***5.50*** ± 0.47	***5.49*** ± 0.57	***5.28*** ± 0.41	*1.0*	*0.02* ^e^
Insulin (pmol/L)^b^	***36.5*** [25.8; 51.6]	***43.5*** [30.3; 62.5]	***36.7*** [27.3; 49.1]	*0.05* ^e^	*1.0*
HOMA-IR	***0.72*** [0.52; 1.0]	***0.85*** [0.60; 1.20]	***0.70*** [0.53; 0.94]	*0.06*	*0.8*
HOMAβ	***62.6*** [53.4; 73.5]	***70.4*** [60.0; 82.7]	***67.2*** [57.5; 78.7]	*0.08*	*0.2*
HbA1c (mmol/mol)^a^	***35.3*** ± 4.15	Not relevant^f^	***35.3*** ± 3.73	Not relevant^f^	*1.0*
Systolic blood pressure mmHg^h^	***112*** ± 11.8	***115*** ± 11.2	***116*** ± 9.16	*0.3*	*0.1*
Diastolic blood pressure mmHg^h^	***71.0*** ± 8.47	***72.3*** ± 8.88	***74.3*** ± 7.01	*0.3*	*0.04* ^e^
Hs-CRP (mg/L)^b^	***1.04*** [0.68; 2.45]	***1.94*** [0.96; 3.15]	***1.21*** [0.64; 2.15]	*0.08*	*0.4*
CD163 (mg/L)	***1.66*** [1.27; 2.10]	***2.33*** [1.58; 2.69]	***1.71*** [1.40; 2.22]	*0.3* ^e^	*0.2*
CD206 (mg/L)	***0.21*** [0.17; 0.25]	***0.23*** [0.20; 0.32]	***0.23*** [0.20; 0.28]	*0.007* ^e^	*0.02* ^e^
MetS^i^	***0 (0%)***	***0 (0%)***	***2 (15%)***	*1.0*	*0.1* ^d^
FSH (IU/L)	***3.9*** [3.0; 22.1]	***68*** [50.4; 93.8]	***39*** [24.2; 50.3]	*0.002* ^e^	*0.03* ^e^
LH (IU/L)	***4.7*** [3.0; 36.1]	***41*** [36.2; 54.2]	***25*** [16.3; 28.8]	*0.004* ^e^	*0.2*
Estradiol (pmol/L)	***471*** [124; 752]	***15*** [15; 25.5]	***27*** [15; 252]	*0.002* ^e^	*0.07*

The italic values reflect P-values, and the bolditalic values reflects means/medians

*BMI* body mass index, *LDL* low-density lipoprotein, *HDL* high-density lipoprotein, *HbA1c* hemoglobin A1c, *Hs-CRP* high sensitivity C-reactive protein, *MetS* metabolic syndrome, *FSH* follicle-stimulating hormone, *LH* luteinizing hormone

^a^Mean

^b^Median

^c^Paired T-test for means and Wilcoxon signed rank test for medians

^d^Pearson chi^2^ test

^e^Statistical significant

^f^During treatment HbA1c was not a relevant measure because hemoglobin is affected by the chemotherapy

^g^n = 11

^h^n = 12

^i^At least 3 of the following 5: HDL < 1.3, triglycerides ≥ 1.7, waistline > 88 cm, fasting glucose ≥ 6.1, blood pressure ≥ 130/85

From T0 to T1 triglycerides increased and HDL decreased significantly (Table 4), and triglycerides remained significantly higher at T2 compared to baseline values. LDL did not change significantly, irrespective of the time interval measured.

From T0 to T1 the insulin level of the premenopausal women increased statistically significant. After 1 year the insulin levels had returned to baseline values (Table 4). Fasting glucose remained stable from T0 to T1, and decreased significantly from T1 to T2. There was no change in HOMA-IR and HOMAβ. During follow up diastolic blood pressure increased significantly (Table 4). Hs-CRP was unaltered, whereas both CD163 and CD206 increased significantly during treatment with chemotherapy. CD206 remained significantly increased 1 year after ended chemotherapy.

Among premenopausal BC patients FSH increased statistically significant during chemotherapy and remained significantly elevated compared to baseline values. Similarly LH increased significantly from T0 to T1, and remained elevated at T2, while estradiol dropped significantly from T0 to T1 and remained at a reduced level at T2 (Table 4).

### Changes in body composition, metabolic parameters and female hormones in all postmenopausal BC patients

In the postmenopausal group of BC patients, we found a significant increase in waist circumference from T0 to T2, while weight, body fat and lean body mass was unchanged (Table 5). Bone mineral density of the postmenopausal women increased significantly from baseline to 1 year post-treatment.

**Table 5:** Changes in parameters related to the metabolic syndrome and females sex hormones in *postmenopausal* BC patients during adjuvant anticancer treatment

	Mean/median			Paired t-test/signed rank^c^	
	T0	T1	T2	T0 vs. T1	T0 vs. T2
Weight (kg)^a^	***76.4*** ± 16.2	***76.7*** ± 15.5	***75.9*** ± 15.6	0.6	0.3
BMI (kg/m^2^)^b^	***26.3*** [22.7; 30.8]	***27.1*** [23.5; 30.8]	***25.8*** [23.1; 30.6]	*0.2*	*0.2*
Waist (cm)^a, g^	***88.7*** ± 11.1	***90.1*** ± 10.6	***91.7*** ± 10.9	*0.1*	*0.03* ^e^
Body fat (%)^a^	***38.5*** ± 5.77	***38.0*** ± 5.65	***38.6*** ± 5.04	0.2	0.8
Lean body mass (kg)^a^	***43.5*** ± 6.13	***44.2*** ± 5.53	***43.2*** ± 6.25	0.07	0.7
Bone mineral density (g/cm^2^)	***1.08*** ± 0.11	***1.08*** ± 0.09	***1.09*** ± 0.09	0.4	0.05^e^
Total cholesterol (mmol/L)	***5.56*** ± 0.83	***5.54*** ± 1.03	***5.73*** ± 0.92	*0.9*	*0.4*
LDL (mmol/L)^a^	***3.24*** ± 0.91	***3.24*** ± 0.99^h^	***3.43*** ± 1.01	*1.0*	*0.3*
HDL (mmol/L)^a^	***1.73*** ± 0.52	***1.45*** ± 0.38	***1.64*** ± 0.44	< 0.0001^e^	*0.05* ^e^
Triglyceride (mmol/L)^b^	***1.15*** [0.9; 1.2]	***1.30*** [1.11; 1.87]	***1.15*** [1.0; 1.39]	*0.006* ^*e*^	*0.2*
Glucose (mmol/L)^a^	***5.90*** ± 0.61	***5.83*** ± 0.88	***5.62*** ± 0.51	0.5	*0.02* ^e^
Insulin (pmol/L)^b^	***41.1*** [32.0; 52.8]	***38.0*** [29.2; 49.6]	***38.6*** [30.5; 49.0]	*0.3*	*0.6*
HOMA-IR	***0.83*** [0.66; 1.04]	***0.76*** [0.59; 0.98]	***0.75*** [0.59; 0.95]	0.2	*0.3*
HOMAβ	***59.4*** [52.7; 67.0]	***57.9*** [51.9; 64.6]	***60.0*** [51.9; 69.4]	0.6	*0.9*
HbA1c (mmol/mol)^a^	***37.0*** ± 4.64	Not relevant^f^	***36.2*** ± 4.73	Not relevant^f^	*0.4*
Systolic blood pressure mmHg^a^	***119*** ± 11.3	***115*** ± 11.4	***116*** ± 10.2	*0.1*	*0.1*
Diastolic blood pressure mmHg^a^	***72.4*** ± 5.87	***70.0*** ± 6.96	***72.3*** ± 6.68	*0.1*	*0.9*
Hs-CRP (mg/L)^b^	***1.43*** [0.76; 3.90]	***1.41*** [1.01; 4.73]	***1.12*** [1.01; 1.70]	*0.8*	*0.01* ^*e*^
CD163 (mg/L)	***1.93*** [1.77; 2.08]	***1.99*** [1.68; 2.40]	***1.93*** [1.67; 2.32]	*0.5*	*0.6*
CD206 (mg/L)	***0.23*** [0.18; 0.29]	***0.28*** [0.23; 0.32]	***0.24*** [0.18; 0.27]	*0.1*	*0.4*
MetS^i^	***5 (25%)***	***3 (15%)***	***3 (15%)***	*0.4*	*0.4* ^d^
FSH (IU/L)	***74.5*** [62.2; 98.4]	***72*** [64.2; 88.7]	***73.5*** [61.5; 87.8]	*0.3*	*0.09*
LH (IU/L)	***41.5*** [36; 44.9]	***39*** [30.7; 42.9]	***32.5*** [30.1; 38.8]	*0.5*	*0.04* ^e^
Estradiol (pmol/L)	***15*** [15; 22.7]	***15*** [15; 18.6]	***15*** [15; 18.3]	*0.3*	*0.2*

The italic values reflect P-values, and the bolditalic values reflects means/medians

*BMI* body mass index, *LDL* low-density lipoprotein, *HDL* high-density lipoprotein, *HbA1c* hemoglobin A1c, *Hs-CRP* high sensitivity C-reactive protein, *MetS* metabolic syndrome, *FSH* follicle-stimulating hormone, *LH* luteinizing hormone

^a^Mean

^b^Median

^c^Paired T-test for means and Wilcoxon signed rank test for medians

^d^Pearson chi^2^ test

^**e**^Statistical significant

^f^During treatment HbA1c was not a relevant measure because hemoglobin is affected by the chemotherapy

^g^n = 17

^h^n = 18, LDL was not analyzed when triglycerides were above 4.0 mmol/L. For two patients this was the case post chemotherapy (T1)

^**i**^At least 3 of the following 5: HDL < 1.3, triglycerides ≥ 1.7, waistline > 88 cm, fasting glucose ≥ 6.1, blood pressure ≥ 130/85

During chemotherapy, from T0 to T1, HDL decreased significantly, and triglycerides increased significantly. After 1 year (T2), HDL remained significantly decreased, while triglycerides returned to baseline values. LDL did not change significantly.

**Fig. 3:**
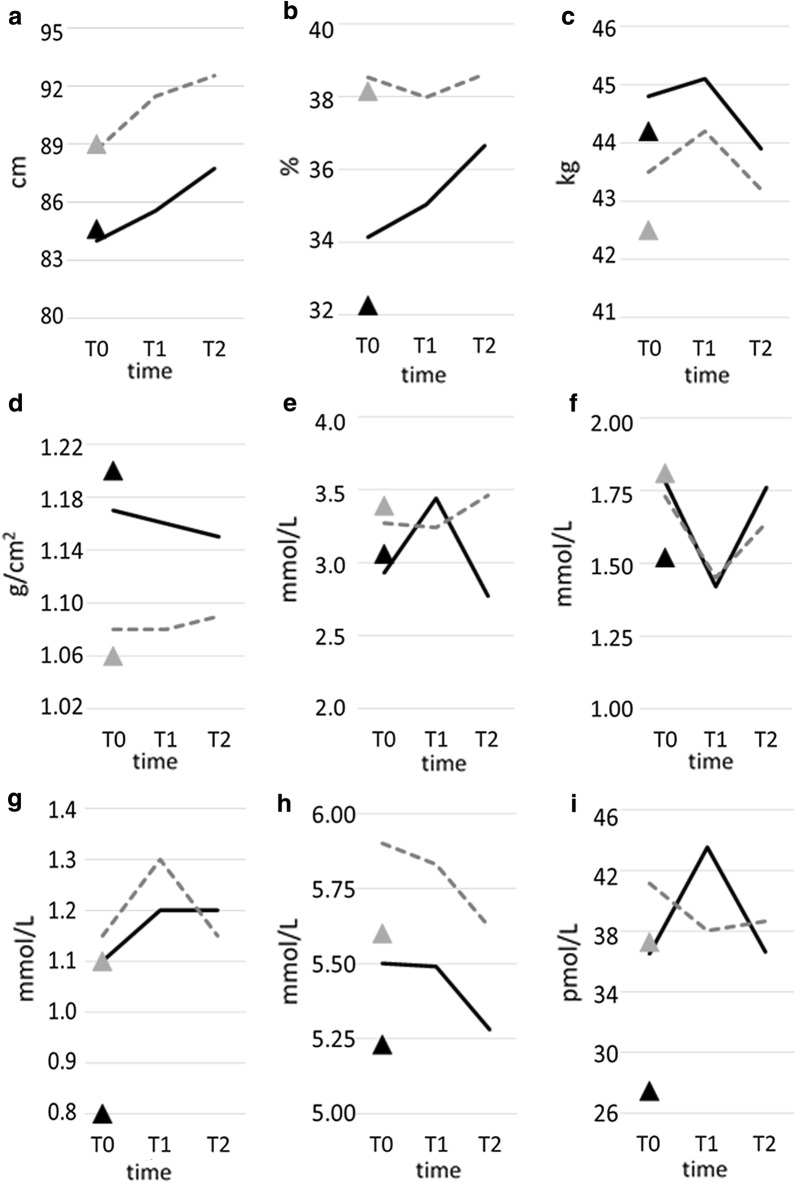
Changes in key parameters during observation period T0-T2 related to menopausal status. **a** Waist, **b** body fat, **c** LBD, **d** BMD, **e** LDL, **f** HDL, **g** triglycerides, **h** glucose, **i** insulin, **j** HOMA-IR, **k** HOMA-β, **l** SBP, **m** DBP, **n** Hs-CRP, **o** MetS. *LBM* lean body mass, *BMD* bone mineral density, *LDL* low-density lipoprotein, *HDL* high-density lipoprotein, *HOMA-IR* homeostatic model assessment of insulin resistance, *HOMA β* homeostatic model assessment of β-cell function, *SBP* systolic blood pressure, *DBP* diastolic blood pressure, *Hs-CRP* high sensitivity C-reactive protein, *MetS* metabolic syndrome. Dotted line, postmenopausal BC patients; straight line, premenopausal BC patients; grey-filled triangle, postmenopausal controls; black-filled triangle, premenopausal controls

Fasting glucose dropped steadily through the follow up period (most pronounced from T1 to T2), (Fig. 3), resulting in an overall significant decrease (Table 5), while insulin, HOMA-IR and HO MAβ were unaltered. Blood pressure was unaltered in postmenopausal women. Hs-CRP decreased significantly in the postmenopausal women during the study period. CD163 and CD206 were unaltered from T0 to T2 in postmenopausal women.

In postmenopausal BC patients FSH was unaltered from T0 to T2, while LH decreased steadily and estradiol was unchanged (Table 5).

### Changes in body composition, metabolic parameters and female hormones in postmenopausal BC patients, who did not receive statins and/or metformin

When excluding the women who received statins and/or metformin we saw small and not clinically convincingly differences; HbA1c was significantly lower in postmenopausal BC patients (38.9 ± 3.50) at baseline compared to postmenopausal controls (36.0 ± 2.65), P = 0.01. Furthermore, we found a small but statistically significant increase in lean body mass from T0 (42.2 ± 5.00) to T1 (43.2 ± 4.73) in the group of postmenopausal women who did not receive any of these treatments, P = 0.01. Finally, we saw a small but statistically significant decline in FSH from T0 (78.0 [65.0;101]) to T2 (75.0 [65.0;89.0]) in this group, P = 0.04. Beside from the three parameters, none of the other results differed from the main analysis (data not shown).

## Discussion

This study shows that specific metabolic changes occur during and after adjuvant chemotherapy and that these changes seem most pronounced among premenopausal women. Looking at pre- and postmenopausal women separately, chemotherapy seemed to lead to greater adverse effects in premenopausal women. Changes were evident in body composition, lipid metabolism, and blood pressure and transiently in glucose homeostasis among premenopausal women, while among postmenopausal women body composition and lipid metabolism was slightly adversely affected. These results are in accordance with a study from Bicakli at al. [21], who also described more pronounced changes in especially body composition among premenopausal BC patients.

At the time of diagnosis of BC both pre- and postmenopausal women had a metabolic profile that was very similar to their respective controls.

Both during adjuvant treatment and the 1st year after premenopausal women experienced a rapid and persistent increase in body fat, primarily around the waist. Body weight and lean body mass did not change significantly in either of the groups. This underlines that weight should be supplemented by measuring waist circumference, when assessing the effects of treatment on body composition. A fat distribution centered around the waist is known to be most disadvantageous, and in combination with loss of muscle mass this may be particularly unfavorable from a metabolic standpoint [22].

Bone mineral density in the postmenopausal women increased significantly during the study period, perhaps as a result of the adjuvant treatment with bisphosphonates (zoledronic acid) and calcium.

A persistent increased level of triglycerides was seen in premenopausal women. Increased levels of triglycerides is known to be related to CVD [23], while HDL and LDL cholesterol changed only transiently and returned to baseline levels during the observation period. How transiently increased levels of cholesterol affect the acute risk of coronary events is still not known. A meta-analyses of 10.864 women followed up for more than 10 years showed, that a triglyceride elevation of 1 mmol/L increased the risk of CVD by 75% in women [23]. This study cannot explain what caused the increase in triglycerides, but suggestively the rise could be caused by the combined induction of menopause in most premenopausal women [11] and treatment with tamoxifen, which is also known to increase the risk of hypertriglyceridemia [17]. In postmenopausal BC patients the observed changes in lipid metabolism were less pronounced compared to premenopausal BC patients, maybe because of zoledronic acids ability to reduce cholesterol biosynthesis via inhibition of the mevalonate pathway [24].

Insulin levels increased slightly and transiently after ended chemotherapy in premenopausal women. Whether this slight increase, which was not mirrored by concomitant changes in fasting blood sugar, HOMA-IR or HOMAβ, was caused by decreasing circulatory estrogen levels or chemotherapy or a combined effect, remains unknown in this study.

Overall, glucose homeostasis remained largely unchanged in both pre- and postmenopausal women. In fact, we observed a slight decrease at the end of the observation period of fasting blood glucose among both pre- and postmenopausal women. At baseline HbA1c was lower among postmenopausal BC patients compared to controls, this difference was statistically significant when excluding the postmenopausal women, who received statins and/or metformin. This finding could indicate that the significant decrease in fasting glucose during follow up (seen in both pre- and postmenopausal patients), is really due to a transient elevated level at baseline, possibly because of surgery [25].

We only used rather crude measures of glucose homeostasis like fasting glucose, insulin and the HOMA index of insulin resistance and beta cell function, and it may be that we would have been able to find more subtle differences between BC patients and controls, had we employed the hyperinsulinemic euglycemic clamp technique. Although it has been shown that the HOMA-IR correlates quite well with the measure of insulin resistance derived from the clamp technique [26].

The effect of treatment on blood pressure was also dependent on menopausal status. Premenopausal women had a significant increase in diastolic blood pressure during the study period, adding to an increased risk of MetS.

The inflammatory response in premenopausal BC patients, expressed by CD163 and CD206, was more pronounced compared to the postmenopausal patients, and signs of inflammation was still evident 1 year after ended chemotherapy among these younger women. Contrary to these findings, the inflammation marker hs-CRP did not increase significantly and remained largely unchanged during the study, despite increase in other variables related to the metabolic syndrome. As part of their treatment, BC women received fairly large amounts of corticosteroids, which is known to have anti-inflammatory effects [27], which could explain the largely unchanged levels. Among postmenopausal women we observed a slight but significant decrease from T0 to T2. This could indicate that breast surgery induces a low grade inflammatory state, as seen in other types of surgery [28], and that this state is maintained during treatment with chemotherapy. The levels of hs-CRP, CD163, and CD206 at baseline though, were not significantly different from that observed among controls.

FSH levels were significantly lower in the premenopausal BC patients at baseline compared to the healthy controls, possibly because of negative feedback from a (non-significantly) higher level of estradiol.

The significant decrease in LH (T0–T2) in postmenopausal women is in accordance with findings from previous studies [10]. But little is known about the hormonal changes after adjuvant chemotherapy, especially in postmenopausal women.

Changes in levels of FSH and LH can also be seen in relation to very emotional and stressful experiences [29].

During treatment female hormones of the premenopausal patients changed significantly and in accordance with induction of menopause. Only few of the premenopausal women can be expected to return to baseline values of FSH, LH and estradiol, and only a longer follow up period would show, which individuals could normalize ovarian function.

All of the above findings could suggest that premenopausal women treated for BC, perhaps augmented by either permanent or transient induction of premature menopause, are more susceptible to changes induced by chemotherapy. The mechanisms behind could be the rapid decline in estradiol, leading to weight gain and central obesity, the last known as the major driving force behind changes related to MetS.

Besides chemotherapy induced menopause, treatment with prednisolone could be part of the explanation for the slight and transient increase in insulin levels among premenopausal women, where even treatment with low dose prednisolone is known to affect glucose metabolism [30].

When excluding the women who received statins and/or metformin from the main analysis, we saw small and not clinically convincingly differences. Larger cohorts would be necessary to conclude whether or not treatment with these medications actually affects the results, and in general how the different treatment modalities influences various outcomes.

The women in our study were informed about their results after each investigation, and had the possibility to act on these results. Today, metabolic control is not a part of standard BC guidelines (http://www.dbcg.dk/PDF%20Filer/Kap_9_Opfoelgning_og_kontrol-11.12.2015.pdf, 10.01.19). This could mean that we are underestimating the actual changes, as the patients in this study, may have made some lifestyle changes, because of their knowledge about their metabolic status.

This study has some limitations. The small size of our cohort makes it difficult to draw any firm conclusions. Furthermore, the short follow-up and the heterogeneity of our patient group was a limitation. It would be preferable to, prolong follow up for more than 1 year and to examine patients even before surgery was performed, in order to get a true baseline value for all metabolic parameters. Furthermore, the study design would be strengthened by adding follow up of the healthy controls. Optimally we would have examined the 36 controls by same means and with same time interval as the cases, in order to study the development among controls in metabolic status. However, we expected that it would be very difficult to retain the control group in the study for a 1-year follow-up. Instead we focused on retaining the 33 breast cancer patients in the study over time, letting these women act as their own controls.

The control group was primarily included to study, if the breast cancer patients per se differed from the background population at baseline. We chose to match controls by age and menopausal status to make them as similar as possibly regarding hormonal status, because this status is important in relation to cardiovascular risk.

## Conclusions

In summary, this explorative study shows that treatment with chemotherapy results in significant, but very specific, changes in several parameters related to cardiovascular and metabolic disease. We observed a differential impact on BC patients, determined to a large extent on menopausal status. The changes may have a significant impact on the future health of the BC patients, and this fact calls for more attention on cardiovascular and metabolic control, especially among premenopausal women who have the longest remaining life expectancy and for whom the most pronounced changes were seen.

Based on our findings, larger follow-up studies focusing on the development of the individual components of MetS are needed to determine, the magnitude of these problems among BC patients.

## Abbreviations

BC: breast cancer
AIs: aromatase inhibitors
SERMs: selective estrogen receptor modulators
CVD: cardiovascular disease
MetS: metabolic syndrome
AUH: Aarhus University Hospital
T0: time point 0 (before start of chemotherapy)
T1: time point 1 [after the final treatment (within 2 weeks after the completion of chemotherapy)]
T2: time point 2 [1 year (11–13 months) after completed chemotherapy]
LDL: low-density lipoprotein
HDL: high-density lipoprotein
HOMA-IR: homeostatic model assessment of insulin resistance
HOMA β: homeostatic model assessment of β-cell function
FSH: follicle-stimulating hormone
LH: luteinizing hormone
hs-CRP: high sensitivity C-reactive protein
DEXA: dual-energy X-ray absorptiometry
LBM: lean body mass
BMD: bone mineral density
BMI: body mass index
SBP: systolic blood pressure
DBP: diastolic blood pressure
